# Characterization of complete mitochondrial genome of *Hylarana guentheri* (Anura: Ranidae) and its phylogenetic implication

**DOI:** 10.1080/23802359.2019.1711216

**Published:** 2020-01-14

**Authors:** Lichun Jiang, Bingxiu Wu, Jing Luo, Zhongwen Xu, Nenghao Huang

**Affiliations:** a Key Laboratory for Molecular Biology and Biopharmaceutics, School of Life Science and Technology, Mianyang Normal University, Mianyang, P.R. China;; b Ecological Security and Protection Key Laboratory of Sichuan Province, Mianyang Normal University, Mianyang, P.R. China

**Keywords:** Hylarana guentheri, ranidae, mitochondrial genome, genome organization, phylogenetic status

## Abstract

The Günther’s frog (*Hylarana guentheri*) belongs to a member of the family Ranidae. We provide a complete mitogenome of *H. guentheri* and examine its phylogenetic position with other related species. Its mitogenome is a closed circular molecule 18,698 bp in length including 13 protein coding genes, 22 tRNA coding genes, two rRNA-coding genes, and a control region (CR) that are conserved in most Ranidae mitogenomes. The overall base composition of the *H. guentheri* mitogenome is 29.27% A, 30.45% T, 26.14% C, and 14.15% G, which is typical for Amphibious animals’ mitochondrial genomes. The alignment of the Ranidae species control regions showed high levels of genetic variation and abundant AT content. Seven tandem repeats were found in the control region. Phylogenetic analysis with Bayesian inference and maximum likelihood based on 13 protein-coding genes indicated that *H. guentheri* is more closely related to *Nidirana okinavana* than to *Babina subaspera* and *B. holsti*. The complete mitogenome of *H. guentheri* provides a potentially useful resource for further exploration of the taxonomic status and phylogenetic relationships of *Hylarana* and related species.

The Günther’s frog (*Hylarana guentheri*) belongs to the family Ranidae. It has mainly distributed in Southeast Asia, Hong Kong, Macau, Taiwan, and Viet Nam (Lue et al. 2009). Although the complete mitochondrial genome analyses have achieved rapid advancement and applied in an increasing number of organisms, its widespread application should not be ignored because it is a multipurpose source of genetic variation for evolutionary study and systematic taxonomic status (Mauro et al. 2004; Huang et al. 2016; Jimenez et al. 2017; Zhang et al. 2018). In recent years, an increasing number of studies have been conducted to expound the molecular phylogenetics in the Neobatrachia (Hahn et al. 2013; Yuan et al. 2016). To better understand the mitochondrial genomic characteristics, phylogeny and evolution of the Ranidae, we determined the complete mitogenomes of *H. guentheri* to provide useful information to species delimitation and taxonomic status studies.

The clipped toe of the frog leg samples were collected from Youxian District, Mianyang City, Sichuan province, China in September 2018 (104°46′44.69″E, 31°29′38.06″N, 484 m.a.s.l) and immediately preserved in 100% ethanol at –70 °C until use. The voucher specimen (LC2018092202) were deposited in the Herbarium of Mianyang Normal University, Mianyang, China. The sample was extracted by phenol-chloride method (Sambrook and David 2001). We employed Long-and-Accurate PCR methods to amplify the whole mitogenomic region of *H. guentheri* with the self-designed and partial universal PCR primers for the mtDNAs of modern frogs (Kurabayashi and Sumida 2009). The whole sequence of the *H. guentheri* mtDNA was determined and deposited to the GenBank DNA databases under accession number MN248533.

The entire mtDNA of *H. guentheri* is a closed circular molecule 18,698 bp in length and comprises 37 genes, 13 protein-coding genes (PCGs), 2 ribosomal RNA genes (12S rRNA and 16S rRNA), 22 tRNA genes and a control region (D-loop). The mitogenome of *H. guentheri* shows the typical gene content observed in vertebrate mitogenomes (Chen et al. 2005; Kurabayashi et al. 2010). The base composition of the whole mitochondrial genome is 29.27% A, 30.45% T, 26.14% C, and 14.15% G. The arrangement of the gene sequence for *H. guentheri* is similar to other ranid frogs. Most protein-coding genes start with the ATG codon, with the exception of ND2, COX1 and ATP6, where ND2, COX1 and ATP6 start with ATA. Only ND4L has TAA termination codon in protein-coding genes. The 22 tRNA genes ranged in length from 64 to 73 bp. The length of D-loop is 2,758 bp, which between tRNA-Leu and Cytb. Furthermore, seven different tandem repeats were characteristic of the CR region for this species.

Phylogenetic trees were reconstructed using BI and ML analyses, based on the nucleotide dataset. The best-fit GTR + G model was selected in jModelTest 0.1 (Darriba et al. 2012), and yielded identical phylogenetic trees by high node-supporting values, including that 30 reported neobatrachian species (Figure 1). It showed that *H. guentheri* and *Nidirana okinavana* converged on the same branch and they have a close genetic relationship. In conclusion, our study characterized the complete mitogenomes of *H. guentheri*, and determined its systematic classification status, which would facilitate further investigations of molecular evolution, genetic structure and conservation of the species.

**Figure 1.**
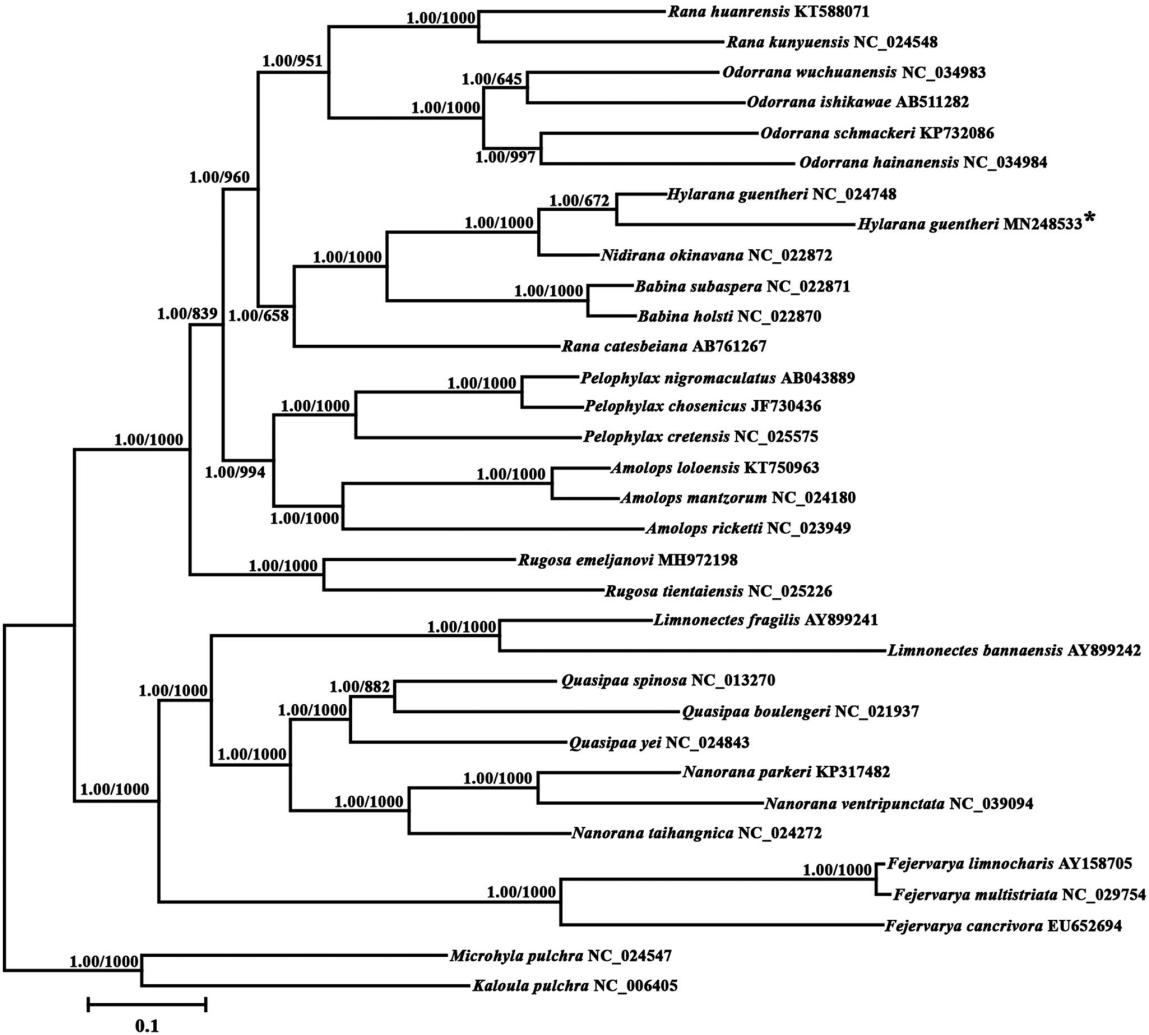
Phylogenetic tree of the relationships among 31 species of Neobatrachia and two species of Microhylidae as outgroups (*Microhyla pulchra* and *Kaloula pulchra*) based on the nucleotide dataset of the 13 mitochondrial protein-coding genes. Branch lengths and topology are from the BI analysis. Numbers above branches specify posterior probabilities from Bayesian inference (BI) and bootstrap percentages from maximum likelihood (ML, 1000 replications) analyses. Tree topologies produced by Bayesian inferences (BI) and maximum likelihood (ML) analyses were equivalent. Bayesian posterior probability and bootstrap support values for ML analyses are shown orderly on the nodes. The asterisks indicate new sequences generated in this study.
